# Risk Factors for Systemic Inflammatory Response Syndrome Induced by Flexible Ureteroscope Combined with Holmium Laser Lithotripsy

**DOI:** 10.1155/2020/6842479

**Published:** 2020-03-21

**Authors:** Qiwu Mi, Xiangjun Meng, Linghui Meng, Derong Chen, Shaowei Fang

**Affiliations:** ^1^Department of Urology, Dongguan People's Hospital, Dongguan, 523059 Guangdong, China; ^2^Xinxiang Medical University, Xinxiang, 453003 Henan, China

## Abstract

**Objective:**

To investigate the risk factors of systemic inflammatory response syndrome (SIRS) induced by flexible ureteroscope combined with Holmium laser lithotripsy. *Patients and Methods*. The clinical data from 216 consecutive patients who had undergone flexible ureteroscope combined with Holmium laser lithotripsy between August 2015 and May 2019 were retrospectively analyzed. To identify the risk factors of systemic inflammatory response syndrome induced by flexible ureteroscope combined with Holmium laser lithotripsy, the cases were divided into two groups according to whether they developed postoperative SIRS: SIRS group (21 cases) and non-SIRS group (195 cases). Age, gender, body mass index, stone size, surgery time, stone location, hydronephrosis, urine culture, hospital stay, stone-free rate, ureteral access sheath, and diabetes mellitus were collected. Univariate analysis was performed to calculate the potential factors. In order to determine the independence of the various factors, factors that potentially contributed to SIRS were compared between the SIRS group and the non-SIRS group. Furthermore, multivariate logistic regression analysis was used to identify the risk factors of systemic inflammatory response syndrome induced by flexible ureteroscopic lithotripsy.

**Results:**

All patients were successfully treated with flexible ureteroscopic lithotripsy. The incidence of SIRS after flexible ureteroscopic lithotripsy was 9.7%. The univariate analysis demonstrated the potential risk factors of systemic inflammatory response syndrome induced by flexible ureteroscopic lithotripsy were stone size (*p* = 0.002), surgery time (*p* = 0.002), surgery time (*p* = 0.002), surgery time (*p* = 0.002), surgery time (*p* = 0.002), surgery time (*p* = 0.002), surgery time (*p* = 0.002), surgery time (*p* = 0.002), surgery time (

**Conclusions:**

Stone size, surgery time, urine culture, and ureteral access sheath are independent risk factors for SIRS induced by flexible ureteroscopic lithotripsy. Patients with these high-risk factors should be carefully evaluated to reduce systemic inflammatory response syndrome.

## 1. Introduction

Flexible ureteroscopic lithotripsy has become an effective and valued procedure in the treatment of upper urinary tract stones. Although flexible ureteroscopic lithotripsy is a minimally invasive technique, it carries the potential risk of complications.

Systemic inflammatory response syndrome (SIRS) is a common and serious complication associated with flexible ureteroscopic lithotripsy. Senocak et al. reported that the incidence of postoperative infectious complications after flexible ureteroscopic lithotripsy was 8.5% [1]. SIRS not only prolongs hospitalization time but also affects the prognosis of patients [2]. SIRS might further develop into multiple organ dysfunction. Therefore, it is important to identify the risk factors of systemic inflammatory response syndrome induced by flexible ureteroscope lithotripsy.

## 2. Patients and Methods

### 2.1. Ethical Approval and Patient Selection

The study protocol was approved by the Dongguan People's Hospital ethical committee (Dongguan, China), and informed consent to publish details was obtained from all individual participants in this study.

From August 2015 to May 2019, a total of 216 cases with upper urinary tract stones treated by flexible ureteroscopic lithotripsy were retrospectively enrolled in this study. The operations were performed by a surgeon with 10 years of experience following standard procedure. The patient demographics and stone characteristics are reported in Table 1. All cases underwent preoperative intravenous urography, urinary ultrasonography, and computed tomography (CT). The length of stones measured by computed tomography indicated the size of stones. Patients with upper urinary stones (≤2 cm) were finally included in our study. In order to minimize potential bias for evaluating risk factors, patients with severe hydronephrosis, uncontrolled urinary tract infection, severe urethral or ureteral stricture, severe hemorrhagic diseases, and cardiopulmonary insufficiency were excluded.

Urinalysis and urine culture were used to evaluate whether or not the urinary tract infection had merged. Patients with infection would not undergo the surgery until the infection was controlled. In those patients, different antibiotics were used according to the severity of infection. Such antibiotic would also be used prophylactically before surgery. All patients received prophylactic antibiotics 30 minutes before surgery.

No residual fragments or residual fragments < 3 mm were defined as stone free. The outcome was confirmed by performing CT or abdominal plain film examination 2 weeks after surgery.

### 2.2. SIRS Criteria

The presence of SIRS was evaluated after flexible ureteroscopic lithotripsy. SIRS criteria were identified according to the American College of Chest Physicians in 2001 (two or more of the following): (1) temperature > 38°C or <36°C, (2) heart rate > 90 beats per minute, (3) respiratory rate > 20 breaths per minute or arterial carbon dioxide tension PaCO2 < 32 mmHg, and (4) white blood cell count > 12,000 cells/*μ*L or <4,000 cells/*μ*L [3].

### 2.3. Surgical Protocol

A ureteral double J tube was indwelled routinely 2 weeks before surgery to dilate the ureter. The lithotomy position was taken under general anesthesia. Firstly, ureteroscope (F8.0/9.8 Wolf) was used for ureteroscopic examination, and the guide wire was detained to the pelvis, and ureteral access sheath was placed along the guide wire. Flexible ureteroscope (Olympus) entered the renal collecting system through ureteral access sheath, and stones were fragmented with a Holmium laser lithotripsy device (Lumenis Versa Pulse Power Suite). Ureteral stenting (JJ stent) was routinely indwelled for 2 to 4 weeks.

### 2.4. Statistical Analysis

Data were presented as either mean ± standard deviation (SD) or median. Age, BMI, stone size, hospital stay, and surgery time were in normal distribution, and Student's test were used to compare the continuous variables between groups. Chi-squared test or Fisher's exact test was used to compare the categorical variables between groups. Then, multivariate logistic regression analysis was used to identify the independent risk factors for SIRS induced by flexible ureteroscopic lithotripsy. SPSS 17.0 (SPSS, Chicago, IL, USA) was used for statistical analysis. Significance was established at *p* < 0.05. A two-tailed probability of less than 0.05 was considered statistically significant.

## 3. Results

A total of 216 patients were finally included in this study. All patients were successfully treated with flexible ureteroscopic lithotripsy, among whom 9.7% (21/216) developed SIRS. The cases were divided into two groups according to whether they developed SIRS after flexible ureteroscopic lithotripsy: SIRS group (21 cases) and non-SIRS group (195 cases). The clinical and perioperative factors of the SIRS group and the non-SIRS group were compared, as shown in Table 2.

The mean age of the SIRS group was 48.3 ± 11.9 years and that of the non-SIRS group was 47.6 ± 13.7 years, with no significant difference between the two groups (*p* = 0.63). No statistically significant difference was found between groups in terms of gender (*p* = 0.959). Statistical analysis identified no statistically significant difference between the two groups with regard to body mass index (25.1 ± 2.4 vs. 24.5 ± 3.6 kg/m^2^, *p* = 0.783). There was no significant difference in hydronephrosis (*p* = 0.83) and diabetes mellitus (*p* = 0.767) between the SIRS group and the non-SIRS group.

The average stone size in the SIRS group was larger than that in the non-SIRS group, and a significant difference was found between the two groups (1.62 ± 0.29 vs. 1.58 ± 0.25 cm, *p* = 0.002). The surgery time was 58.0 ± 9.1 min in the SIRS group and 57.4 ± 7.6 min in the non-SIRS group. There was significant difference between the two groups (*p* = 0.01). In the SIRS group, 17 cases were performed with 12/14 F ureteral access sheath and 4 cases with 14/16 F ureteral access sheath. In the non-SIRS group, 84 cases were performed with 12/14 F ureteral access sheath and 111 cases with 14/16 F ureteral access sheath. There was significant difference between the two groups (*p* = 0.001). Urine culture (*p* ≤ 0.001) was significantly associated with postoperative SIRS. The patients with SIRS routinely underwent blood culture, there were 8 (38.1%) of 21 patients who had a positive blood culture in the SIRS group. The average hospital stay in the SIRS group was longer than that in the non-SIRS group, and a significant difference was found between the two groups (5.78 ± 0.48 vs. 3.41 ± 0.23 cm, *p* ≤ 0.001), as shown in Table 3.

In univariate analysis, we observed that there were four potential risk factors for systemic inflammatory response syndrome induced by flexible ureteroscopic lithotripsy, including stone size (*p* = 0.002), surgery time (*p* = 0.01), urine culture (*p* ≤ 0.001), and ureteral access sheath (*p* = 0.001). Furthermore, multivariate logistic regression analysis was used to identify the risk factors for systemic inflammatory response syndrome induced by flexible ureteroscopic lithotripsy. In multivariate logistic regression analysis, stone size (*p* = 0.002, OR = 1.618; 95% CI, 0.452-0.844), surgery time (*p* ≤ 0.001, OR = 1.025; 95% CI, 1.016-1.034), urine culture (*p* ≤ 0.001, OR = 25.795; 95% CI, 22.131-30.065), and ureteral access sheath (*p* ≤ 0.001, OR = 6.101; 95% CI, 5.109-7.284) were identified as independent risk factors for SIRS induced by flexible ureteroscopic lithotripsy.

## 4. Discussion

With the development of endourological minimally invasive technology, flexible ureteroscopic lithotripsy has become one of the most important treatment methods for upper urinary tract stones.

For the kidney and the upper ureteral calculi (less than 2 cm), flexible ureteroscopic lithotripsy has the advantages of less traumatic, quicker recovery, and shorter hospitalization time. However, with relatively high incidence of complications, the application of flexible ureteroscopic lithotripsy is limited.

Systemic inflammatory response syndrome is a common and serious complication associated with flexible ureteroscopic lithotripsy. Studies showed that the incidence of postoperative infectious complications after flexible ureteroscopic lithotripsy was 6.7-20.7% [1, 4]. SIRS not only prolongs hospitalization time but also affects the prognosis of patients [2]. SIRS can further develop into multiple organ dysfunction, with a mortality rate is as high as 20% [5, 6]. Therefore, it is very important to identify the risk factors of systemic inflammatory response syndrome induced by flexible ureteroscope lithotripsy.

Sugihara et al. retrospectively analyzed the data of 12,372 cases who underwent ureteroscopic lithotripsy [7]. The results showed that, compared with whose surgery time within 60 minutes, the relative risk of serious complications in patients whose surgery time was less than 90-120 minutes or over 210 minutes was 1.58 or 4.28 times, respectively. Therefore, they thought that there was a positive correlation between complications and surgery time. Surgery time was a risk factor for infective complication, which might increase the probability of retrograde infection via procedure [8]. In our study, logistic regression analysis showed that surgery time (*p* ≤ 0.001, OR = 1.025; 95% CI, 1.016-1.034) was an independent risk factor for systemic inflammatory response syndrome induced by flexible ureteroscope lithotripsy. We believe that with the prolongation of surgery time, the amount of bacteria and endotoxin absorbed into the blood through the reflux or pelvic mucosa gradually increases, they cooperate to contribute to the inflammatory response. Several studies confirmed that the presence of retrograde infection via procedure was associated with risk factors for the occurrence of septic shock [9, 10].

In a study, the data from enrolled 260 patients were retrospectively analyzed, and stone size was identified as independent risk factors for SIRS induced by flexible ureteroscopic lithotripsy [9]. Yang et al. concluded that patients with larger stone size might have a higher risk of developing systemic inflammatory response syndrome and fever after percutaneous nephrolithotomy [11]. In the present study, according to multivariate analysis, stone size is a risk factor for systemic inflammatory response syndrome induced by flexible ureteroscope lithotripsy. Stone with larger size will increase the difficulty of procedures, which may lead to the prolong surgery time and increase the probability of systemic inflammatory response syndrome via procedures.

Senocak et al. retrospectively reviewed data from 492 consecutive patients who had undergone flexible ureteroscope lithotripsy with stone disease, 42 (8.5%) of 492 patients had postoperative infectious complications after flexible ureteroscope lithotripsy, and 59 (12%) of 492 patients had positive preoperative urine culture [1]. It is generally believed that positive preoperative urine culture is a risk factor of systemic inflammatory response syndrome induced by flexible ureteroscope lithotripsy [12–14]. In our study, there were 16 (76.2%) of 21 patients who had a positive urine culture in the SIRS group; we concluded that urine culture (*p* ≤ 0.001) was significantly associated with postoperative SIRS.

Gutierrez et al. and Korets et al. showed that SIRS after flexible ureteroscope lithotripsy could not be avoided, if broad-spectrum antibiotics were given to patients with negative urine, or sensitive antibiotics were given to patients with positive urine culture [15, 16]. Erdil et al. suggested that preoperative positive urine cultures, intraoperative positive renal pelvic urine cultures, and stone cultures were strongly correlated with the development of SIRS (*p* = 0.001) [12]. Study showed that a biofilm forming in calculi made the antibiotics difficult to kill bacteria [17]. During flexible ureteroscope lithotripsy, the bacteria and endotoxin in the stone will be released, which may cause systemic inflammatory response syndrome. Although patients with urinary tract infection were treated with antibiotics before the procedures, 9.7% (21/216) of these patients still developed systemic inflammatory response syndrome in our study. Unfortunately, the clinical data collected in this study did not include routine culture and analysis of calculi and could not evaluate the correlation between the calculi and the occurrence of SIRS. Therefore, for patients with preoperative positive urine culture, anti-infective treatment should be fully given before procedure. During operation, urine and calculi should be routinely retained and cultured. Component analysis of calculi can provide evidence for anti-infection treatment after operation.

Previous studies have shown that irrigation pressure is a risk factor for urinary sepsis after endourological procedures [18, 19]. When the renal pelvic pressure exceeds 30 mmHg, pelvic reflux might be induced. In the case of intrapelvic hyperpressure, the longer the operation time, the more chance and quantity of bacteria or endotoxin can be absorbed through the pelvic reflux [20]. Small-caliber ureteral access sheath was identified as an independent risk factor for systemic inflammatory response syndrome after flexible ureteroscope lithotripsy [9]. Interestingly, in our study, we observed the correlation between ureteral access sheath and systemic inflammatory response syndrome after flexible ureteroscope lithotripsy. We think that small caliber (12/14) may lead to higher pelvic pressure compared to 14/16 F ureteral access sheath. Therefore, a large-caliber ureteral access sheath for better drainage is required to keep a low renal pelvic pressure during flexible ureteroscope lithotripsy.

There were several limitations in our study. First, the preoperative examination of some patients is not perfect, which leads to the lack of possible potential factors. Second, due to technical factors and retrospective nature, our study failed to include irrigation pressure, stone bacterial culture, and stone composition analysis, which might relate to flexible ureteroscope lithotripsy. Finally, this is a small sample of our study, selection bias is prone to occur. It is necessary to use a larger prospective cohort for further research.

## 5. Conclusions

According to the results achieved by a single experienced surgeon, stone size, surgery time, urine culture, and ureteral access sheath are independent risk factors for SIRS induced by flexible ureteroscopic lithotripsy. Patients with these high-risk factors should be carefully evaluated to reduce systemic inflammatory response syndrome.

## Figures and Tables

**Table 1:** Clinical characteristics of study population.

Variables	Mean (SD) or *N* (%)
Patients (*n*)	216
Age (years)	47.8 ± 12.7
BMI (kg/m^2^)	24.7 ± 3.1
Stone size (cm)	1.59 ± 0.52
Surgery time (min)	57.4 ± 13.8
Gender (*n*)	
Male	104 (48.1)
Female	112 (51.9)
Hydronephrosis	87 (40.3)

BMI = body mass index; SD = standard deviation; stone size = the diameter of stone based on preoperative CT scanning.

**Table 2:** Univariate analysis of risk factors for systemic inflammatory response syndrome induced by flexible ureteroscopic lithotripsy.

Variable	SIRS	No SIRS	*p* value
Age (years)	48.3 ± 11.9	47.6 ± 13.7	0.630
Gender			0.959
Male	10 (47.6)	94 (48.2)	
Female	11 (52.4)	101 (51.8)	
BMI (kg/m^2^)	25.1 ± 2.4	24.5 ± 3.6	0.783
Stone size (cm)	1.62 ± 0.29	1.58 ± 0.25	0.002
Stone location			0.941
Ureteral stones	7 (33.3)	70 (35.9)	
Renal stones	9 (42.9)	76 (39)	
Combined procedure	5 (23.8)	49 (25.1)	
Urine culture			≤0.001
Positive	16 (76.2)	19 (16.2)	
Negative	5 (23.8)	176 (83.8)	
Ureteral access sheath			0.001
F12/14	17 (81)	84 (46.8)	
F14/16	4 (19)	111 (53.2)	
Surgery time (min)	58.0 ± 9.1	57.4 ± 7.6	0.01
Hydronephrosis			0.830
Yes	8 (38.1)	79 (40.5)	
No	13 (61.9)	116 (59.5)	
Diabetes mellitus			0.767
Yes	2 (9.5)	15 (7.7)	
No	19 (90.5)	180 (92.3)	
Hospital stay (d)	5.78 ± 0.48	3.41 ± 0.23	≤0.001
Stone-free rate (%)	85.7	89.4	0.570

BMI = body mass index; SIRS = systemic inflammatory response syndrome; stone size = the diameter of the largest stone based on preoperative CT scanning.

**Table 3:** Multivariate logistic regression analysis of risk factors for systemic inflammatory response syndrome induced by flexible ureteroscopic lithotripsy.

Factors	*B*	Wals	*p* value	OR	95% CI
Stone size (cm)	-0.482	9.163	0.002	1.618	0.452-0.844
Urine culture	3.250	1729.413	≤0.001	25.795	22.131-30.065
Surgery time	0.025	29.310	≤0.001	1.025	1.016-1.034
Ureteral access sheath	1.808	399.587	≤0.001	6.101	5.109-7.284

OR = odds ratio; CI = confidence interval.

## Data Availability

The data used to support the findings of this study are available from the corresponding author upon request.

## Abbreviations

SIRS: Systemic inflammatory response syndrome
BMI: Body mass index
SD: Standard deviation
CT: Computed tomography
OR: Odds ratio
CI: Confidence interval.
